# Efficacy of Intravascular Therapeutic Hypothermia for Moderate to Severe Hypoxic–Ischemic Encephalopathy

**DOI:** 10.3390/children12050605

**Published:** 2025-05-06

**Authors:** Tomonori Kurimoto, Takuya Tokuhisa, Itaru Hayasaka, Tsuyoshi Yamamoto, Eiji Hirakawa, Hiroshi Ohashi, Masaya Kibe, Asataro Yara, Takatsugu Maeda, Masato Kamitomo, Satoshi Ibara

**Affiliations:** 1 Department of Neonatology, Perinatal Medical Center, Kagoshima City Hospital, Kagoshima 890-8760, Japan; tokutaku0305@nifty.com (T.T.); itaru-hayasaka@khc.biglobe.ne.jp (I.H.); yamatuyoshi1@me.com (T.Y.); hirakawaeiji@gmail.com (E.H.); hiroshioohashi12357@gmail.com (H.O.); doc.mkibe@gmail.com (M.K.); morning.bell514@gmail.com (A.Y.); ibara-s40@kch.kagoshima.jp (S.I.); 2 Department of Pediatrics, Social Medical Corporation Bokoi, Nikko Memorial Hospital, Muroran 051-8512, Japan; 3 Department of Obstetrics and Gynecology, Perinatal Medical Center, Kagoshima City Hospital, Kagoshima 890-8760, Japan; maeda-t94@kch.kagoshima.jp (T.M.); mk890054@gmail.com (M.K.)

**Keywords:** hypoxic–ischemic encephalopathy, therapeutic hypothermia, intravascular cooling, extracorporeal membrane oxygenation, neurodevelopment, Apgar score, severe acidosis, persistent pulmonary hypertension of the newborn

## Abstract

Background/Objectives: Hypoxic–ischemic encephalopathy (HIE), affecting 1.3–1.7/1000 live births, is treated with conventional therapeutic hypothermia (TH) but carries significant mortality and neurological impairment. Here, we compared intravascular cooling with extracorporeal membrane oxygenation (ECMO) and conventional TH in neonates with moderate to severe HIE. Methods: We retrospectively analyzed single-center neonates born in 2000–2022. Neonates with a 10 min Apgar score ≤ 3 or umbilical artery pH ≤ 6.7, along with persistent pulmonary hypertension of the newborn and an oxygenation index of ≥25 to <40, were divided into ECMO (*n* = 17) and conventional TH (*n* = 18) groups and administered the Kyoto Scale of Psychological Development at 18 months. Results: Neonatal and maternal characteristics were similar between the groups. A significantly higher proportion of the ECMO group (70.6% vs. 33.3%) achieved a developmental quotient ≥ 70. Conclusions: Intravascular cooling with ECMO may improve the neurodevelopmental outcomes of neonates with HIE, severe acidosis, and low Apgar scores.

## 1. Introduction

Perinatal hypoxic–ischemic encephalopathy (HIE) has an incidence of 1.3–1.7 per 1000 live births [1]. Despite therapeutic hypothermia (TH) being the standard treatment for moderate to severe HIE, numerous studies have reported significant mortality or severe neurological impairment rates by 18 months of age. Previous studies, including the Cool Cap trial, Eunice Kennedy Shriver National Institute of Child Health and Human Development, Rockville, Maryland (NICHD) trial, Total Body Hypothermia (TOBY) trial, and Baby Cooling Japan study, have demonstrated mortality or severe disability rates of 29.5–54.6% [2,3,4,5].

Among neonates undergoing TH, those with an umbilical artery pH ≤ 6.7 frequently develop severe neurological impairment, and seizures have been reported in up to 80% of such cases [6,7]. A 10 min Apgar score ≤ 3 is also strongly associated with adverse long-term outcomes, with 63.2% of affected infants experiencing moderate to severe neurological impairments or mortality by 6–7 years of age [8]. In the NICHD trial, 22% of neonates with moderate to severe HIE had persistent pulmonary hypertension of the newborn (PPHN), which was associated with higher severe encephalopathy and mortality rates [9]. Based on these findings, we studied neonates who underwent TH for HIE at our institution. We specifically focused on those with an umbilical artery pH ≤ 6.7 or a 10 min Apgar score ≤ 3 in combination with PPHN. Outcomes were compared between infants treated with intravascular cooling combined with extracorporeal membrane oxygenation (ECMO) and those who received conventional TH alone. This study is a retrospective chart review conducted at a single tertiary care center, not a randomized controlled trial.

## 2. Materials and Methods

### 2.1. Study Outline and Target Group

This retrospective study included neonates who were admitted to the neonatal intensive care unit at Kagoshima City Hospital, Japan, between January 2000 and October 2022. The Clinical Research Review Board of Kagoshima City Hospital approved the study (approval no. 2024-06). All procedures were conducted in compliance with the ethical standards outlined in the TOBY guidelines and the Baby Cooling Registry of Japan [5,10,11].

### 2.2. Inclusion and Exclusion Criteria

The indications for TH were the following: (i) gestational age ≥ 36 weeks, birth weight ≥ 1800 g, 10 min Apgar score ≤ 5, blood pH ≤ 7.00 or base deficit > 16 mmol/L within the first hour of life, or requirement for assisted ventilation for ≥10 min during resuscitation; (ii) clinical features consistent with moderate to severe HIE (Sarnat stage II or III), such as lethargy, stupor, or coma, accompanied by signs including hypotonia, abnormal reflexes (e.g., pupillary or oculomotor dysfunction), a weak or absent suck reflex, or clinical seizures; (iii) abnormalities observed in the amplitude-integrated electroencephalogram (aEEG) confirmed before TH initiation, persisting for at least 30 min such as flat tracing, low voltage, burst–suppression patterns, or seizure activity; (iv) commencement of TH within 6 h of birth; (v) parental consent provided for the procedures.

We included neonates diagnosed with persistent pulmonary hypertension of the newborn (PPHN), defined by an oxygenation index (OI) between 25 and 40 and supported by consistent clinical signs and echocardiographic findings, including the absence of structural heart disease, evidence of elevated pulmonary arterial pressure, and/or a flattened interventricular septum [9,12]. Infants who met the exclusion criteria—such as presence of chromosomal abnormalities, congenital heart disease, or intracranial hemorrhage, initiation of TH after 6 h of life, or gestational age < 35 weeks—were excluded from analysis.

### 2.3. Study Groups

**Figure 1:**
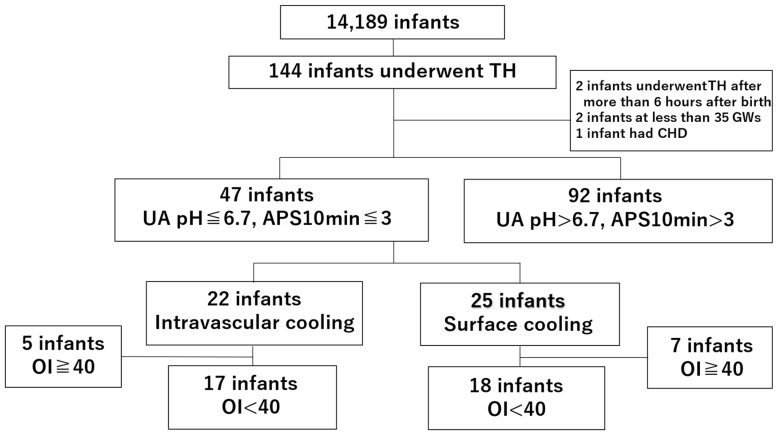
Study flow diagram of neonates undergoing therapeutic hypothermia. TH, therapeutic hypothermia; GWs, gestational weeks; CHD, congenital heart disease; UA, umbilical artery; APS, Apgar score; OI, oxygenation index.

Of the 14,189 infants reviewed, 144 underwent TH for HIE. Five infants were excluded based on the following criteria: TH initiated after 6 h of life (n = 2); gestational age < 35 weeks (n = 2); and congenital heart disease (aortopulmonary window, coarctation of the aorta, and a ventricular septal defect; n = 1). A total of 47 neonates met the following criteria: umbilical artery or neonate’s venous blood pH ≤ 6.7 or a 10 min Apgar score ≤ 3, and PPHN. The OI of 35 cases was ≥25 but <40 [6,7,8,12]. Thus, a cohort of 17 neonates treated with intravascular cooling and ECMO was compared to a cohort of 18 neonates treated with conventional TH (Figure 1). Importantly, the two groups appeared similar at baseline in terms of Apgar scores, umbilical artery pH, and abnormal aEEG prior to the initiation of therapeutic hypothermia, which supports the comparability of the hypoxic injury between the groups.

Developmental assessments using the Kyoto Scale of Psychological Development, a standardized developmental test for Japanese children, were performed at 18 months of age by trained psychologists [13,14].

### 2.4. Conventional Therapeutic Hypothermia (Surface Cooling Method)

The neonates’ head and body temperatures were independently regulated using a cooling blanket (Medi-Therm 2 Hyper/Hypothermia Machine, MTA 5900 Series; Gaymar Industries, Orchard Park, NY, USA). An esophageal temperature probe (Mon-a Therm™ General Sensor; Medtronic, Minneapolis, MN, USA) was inserted into the lower one-third to one-quarter of the esophagus with placement confirmed via radiographic imaging. The esophageal temperature was carefully controlled and maintained at 33.5 °C. The TH was commenced within the first 6 h after birth and sustained for 72 h. Whole-body cooling was achieved using a cooling blanket, with the core temperature reduced to and maintained at 33.5 °C for the 72 h duration of TH. Rewarming was performed gradually at a rate of 0.5 °C/h until a temperature of 37 °C was reached.

### 2.5. Intravascular Therapeutic Hypothermia (Intravascular Cooling with ECMO)

Intravascular cooling was performed using ECMO in neonates with an OI of 25–40 [12]. Although additional parameters such as cord blood pH, base excess, lactate levels, and Apgar scores at 5 and 10 min were considered in the clinical decision-making process, the formal inclusion criterion for this retrospective study was based solely on moderate persistent pulmonary hypertension of the newborn (PPHN) with an OI of 25–40. Intravascular cooling with ECMO initiation followed the institutional protocol, which defined both inclusion and exclusion criteria, as detailed in Supplementary Table S1. A catheter (Fem-Flex, 8–12 F; Edwards Life Sciences, Irvine, CA, USA) was inserted into the right internal jugular vein and advanced cephalad to the internal jugular sinus for ECMO. Whole-body hypothermia was achieved by circulating the cooled blood back into the neonate. The pump cooling flow rate was initiated at 20 mL/kg/min and adjusted between 20 and 40 mL/kg/min during venovenous (VV) ECMO. The low-flow VV ECMO approach (20–40 mL/kg/min) was used as a bridge for oxygenation and controlled cooling rather than full cardiopulmonary support, reflecting an institutional protocol during this period. The goal was to maintain minimally invasive support while achieving sufficient cooling and oxygenation in neonates with moderate PPHN (OI 25–40).

The temperature of the internal jugular sinus was monitored by blood sample withdrawal through the catheter. The targeted temperature was set at 34.0 °C, and the temperature was maintained within a controlled range throughout the 72 h hypothermia phase. Based on previous institutional data, a temperature of 34.0 °C was used to balance neuroprotection and safety [15]. Thereafter, rewarming was conducted gradually at a rate of 0.5 °C/h until the core temperature reached 37 °C. The jugular temperature was typically achieved within 30 to 60 min after ECMO initiation. For comparison, in the surface cooling group, the target temperature was reached in 60 to 120 min.

Although the baseline neurological severity, including Apgar scores, umbilical artery pH, and aEEG findings, was similar between the intravascular and the conventional TH groups, the decision to initiate ECMO was based on additional clinical factors. Specifically, neonates in the intravascular cooling group exhibited persistent hypoxemia and hemodynamic instability despite maximal ventilatory and pharmacologic support. ECMO was initiated when the attending neonatologist judged that surface cooling alone would be insufficient to maintain adequate oxygenation and circulatory function. Treatment decisions were made by the attending neonatologists based on clinical severity and oxygenation status. In some cases, ECMO initiation was also affected by real-time resource availability (e.g., machine access, ECMO-trained staff) and evolving institutional protocols during the study period. These reflect real-world constraints in a tertiary NICU and may have influenced group assignment in this retrospective analysis.

### 2.6. Blood Sample pH ≤ 6.7 Within 60 min After Birth and 10 min Apgar Score ≤ 3

Studies have shown that seizures occur in a notably high proportion—approximately 80%—of neonates with an umbilical arterial blood pH of 6.61–6.70 [7]. Infants with severe neurological impairment after TH often have a significantly low umbilical artery pH (around 6.85 ± 0.15) [6,7]. Thus, a blood pH threshold of 6.7 was established as the decisive cutoff value. A 10 min Apgar score ≤ 3 is also strongly associated with adverse long-term outcomes, with 63.2% of such infants developing moderate to severe neurological impairment or dying by 6–7 years of age [8]. Therefore, this criterion was used for study inclusion.

### 2.7. Data Collection

The collected data included maternal characteristics, fetal heart rate (FHR) monitoring results, and neonatal characteristics. An abnormal FHR was evaluated according to the 2009 guidelines established by the NICHD [16,17,18]. We defined late decelerations as recurrent (occurring in ≥50% of uterine contractions) and bradycardic (FHR < 110 bpm lasting for ≥10 min) [19].

### 2.8. Statistical Analysis

Intergroup comparisons were performed using Chi-square and Fisher’s exact tests. As the continuous variables were non-normally distributed, they were analyzed using the Mann–Whitney U test. Statistical significance was defined as *p* < 0.05, with 95% confidence intervals. All statistical analyses were performed using the R software (version 4.3.1; R Foundation for Statistical Computing, Vienna, Austria) and JMP 14 (SAS Institute, Inc., Cary, NC, USA).

## 3. Results

At 18 months, a significantly greater proportion of infants in the intravascular cooling group achieved a developmental quotient (DQ) ≥ 70 on the revised Kyoto Scale of Psychological Development (12 of 17; 70.6%) compared to the conventional TH group (33.3%) (Table 1). No major ECMO-related complications, such as cannulation site bleeding, infection, or circuit failure, were reported in this cohort.

## 4. Discussion

In this study, intravascular cooling with ECMO improved the outcomes of neonates with hypoxic–ischemic encephalopathy who presented with severe acidosis (umbilical artery pH ≤ 6.7) or critical Apgar scores (≤3 at 10 min) and persistent pulmonary hypertension of the newborn (PPHN). A significantly higher proportion of infants in the intravascular cooling group achieved a developmental quotient of ≥70 at 18 months compared to those treated with conventional therapeutic hypothermia. Although the cesarean section rate was significantly higher in the conventional TH group than in the intravascular cooling group, the underlying obstetric indications—such as placental abruption, non-reassuring fetal status, umbilical cord prolapse, shoulder dystocia, uterine rupture, and malpresentation—did not differ between the groups. Furthermore, there were no significant differences in neonatal Apgar scores (1, 5, or 10 min), umbilical artery pH, or base excess. These findings suggest that the difference in the cesarean section rate did not reflect a difference in illness severity or perinatal compromise between the groups.

The application of ECMO in this high-risk population may offer dual benefits—managing severe hypoxemia and enabling rapid and stable cooling—potentially reducing the neurological injury. However, the retrospective design and limited sample size of our study restrict the generalizability of these findings. Larger, multicenter prospective studies are warranted to validate the efficacy of this approach and assess its long-term neurodevelopmental impact. A randomized controlled trial conducted in the United Kingdom (Neonatal ECMO Study of Temperature) included children with severe cardiopulmonary failure who required ECMO [20]. Although no significant intergroup differences were observed in mortality or neurodevelopmental outcomes at two years, this study did not target HIE cases, and 92–94% of the included infants had 5 min Apgar scores ≥ 5. An analysis of the Extracorporeal Life Support Organization database (2005–2013) compared neonates with HIE who received ECMO with or without whole-body cooling. No significant differences were found in complications or mortality [21]. However, many cases lacked Sarnat staging or were classified as mild.

A single-center study of 20 neonates with moderate to severe HIE and PPHN requiring ECMO (OI > 40) reported outcomes after conventional TH. Nevertheless, the absence of a control group limited the conclusions regarding the added value of ECMO cooling [22]. To date, no study has directly compared intravascular cooling with conventional TH for neonates with moderate to severe HIE and PPHN.

In our study, ECMO was initiated at a pump flow rate of 20 mL/kg/min and maintained at 20–40 mL/kg/min during VV ECMO for cases with an OI of 25–40. The favorable neurodevelopmental outcomes observed may be due to the rapid induction of core hypothermia, improved oxygenation, and more stable temperature control. Although neonates requiring ECMO are often presumed to be more critically ill, our findings suggest that improved circulatory support and precise cooling management may have contributed to the better neurodevelopmental outcomes in the intravascular cooling group. This is further supported by the comparable initial severity profiles between the groups, including similar Apgar scores, pH values, and aEEG abnormalities. This trend toward better outcomes in the intravascular cooling group cannot be explained by differences in baseline characteristics, as no significant differences were observed in gestational age.

In adult cardiac arrest patients, intravascular cooling has been associated with lower temperature variability and more precise temperature maintenance during therapeutic hypothermia [23,24]. ECMO, even with modest flow rates (e.g., 20 mL/kg/min), may help correct hypoxia and acidosis, support pulmonary rest, and maintain systemic oxygenation. These effects, when combined with better therapy control, may contribute to improved neuroprotection.

While intergroup comparisons were conducted in this study, we acknowledge the importance of evaluating subgroup-specific outcomes in future research. Further prospective studies focusing on within-group trends and stratified analyses are warranted.

### Limitations

The retrospective design and single-center setting limit the external validity and generalizability of the findings. Multicenter studies involving larger and more diverse populations are necessary to validate these results. In addition, the sample size—particularly, the number of neonates who underwent intravascular cooling—was relatively small, which reduced the statistical power and may have impacted the precision of the observed associations. Because of the limited sample size, only bivariate analyses were conducted, and potential confounders could not be adjusted for using multivariate statistical methods. Although maternal, fetal, and neonatal factors were taken into account, residual confounding, including unmeasured genetic or environmental influences, may still have been present. Furthermore, genetic testing was not performed; however, no cases of clinically suspected genetic disorders were identified. The decision to initiate ECMO was made based on physician discretion, considering the infant’s clinical stability, resource availability, and institutional protocols at the time. This approach, while reflective of real-world practice, may have introduced selection bias between the groups. In particular, the ECMO flow rate used in this study (20–40 mL/kg/min) is lower than that in standard VV ECMO protocols and was applied as a supportive measure for oxygenation and controlled cooling, rather than for full cardiopulmonary support. This institutional strategy may limit the generalizability of the findings. Notably, no major ECMO-related complications such as bleeding, infection, or circuit failure were reported in this cohort. Finally, the follow-up period was restricted to 18 months, which precluded the evaluation of long-term neurodevelopmental outcomes. Despite these limitations, the study provides preliminary evidence supporting the potential utility of intravascular cooling with ECMO in high-risk neonates, which warrants further investigation in prospective, large-scale studies. This was not a hypothesis-testing RCT; the findings should be considered exploratory due to the limited statistical power.

## 5. Conclusions

Our study findings suggest that intravascular cooling with ECMO may benefit neonates with HIE, severe acidosis, and critical Apgar scores. This approach was associated with improved developmental outcomes compared to conventional TH, supporting the need for prospectives studies to clarify its role in clinical practice.

## Figures and Tables

**Table 1:** Comparison of patient characteristics and clinical outcomes between groups.

	Intravascular TH (% or IQR)	Conventional TH (% or IQR)	*p* Value
	17 (48.6)	18 (51.4)	
Birth weight (g)	2830 (2677–3081)	2923 (2559–326)	0.87
GWs	39 (38–40)	38 (37–40)	0.49
Sex (male)	10 (58.8)	9 (50.0)	0.74
Twin	1 (5.9)	0	0.49
Out-of-hospital birth	17 (100.0)	17 (94.4)	1
Maternal age (years)	36 (27–37)	31.5 (28–35)	0.42
Primipara	10 (58.8)	11 (61.1)	1
PROM	2 (11.8)	1 (5.6)	0.6
HDP	1 (5.9)	3 (16.7)	0.6
GDM	0	2 (11.1)	0.49
HELLP	0	0	
CAM	1 (5.9)	0	0.49
Funisitis	1 (5.9)	0	0.49
Fertility treatment	0	0	
C/S	8 (47.1)	16 (88.9)	0.01
Cervical os bleeding	1 (5.9)	2 (11.1)	1
Placental abruption	10 (58.8)	9 (50.0)	0.74
Shoulder dystocia	1 (5.9)	0	0.49
Umbilical cord prolapse	3 (17.6)	1 (5.6)	0.34
Protracted labor	2 (11.8)	1 (5.6)	0.6
Malpresentation	0	1 (5.6)	1
Ineffective uterine contractions	2 (11.8)	0	0.23
Soft-tissue dystocia	1 (5.9)	0	0.49
Uterine rupture	0	1 (5.6)	1
Umbilical injury	1 (5.9)	0	0.49
NRFS	17 (100.0)	16 (88.9)	0.49
Severe V/Ds	4 (23.5)	4 (22.2)	1
Recurrent L/Ds	6 (35.3)	4 (22.2)	0.47
Severe P/Ds	6 (35.3)	1 (5.6)	0.04
Bradycardia	12 (70.6)	6 (33.3)	0.04
Loss of variability	5 (29.4)	2 (11.1)	0.23
Apgar, 1 min	1 (1–2)	1 (0–2)	0.89
Apgar, 5 min	2 (1–3)	3 (1–4)	0.28
Apgar, 10 min	2 (1–4)	3 (2–5)	0.17
UA pH	6.67 (6.66–6.99)	6.68 (6.57–6.90)	0.41
UA BE	−22.6 (−25.0–−14.6)	−19.6 (−25.7–−13.9)	0.64
Chest compressions (resuscitation)	10 (58.8)	8 (44.4)	0.5
Intubation (resuscitation)	16 (94.1)	12 (66.7)	0.09
Adrenaline (resuscitation)	4 (23.5)	6 (33.3)	0.71
Flat, low voltage (aEEG)	9 (52.9)	9 (50.0)	1
Severe HIE	12 (70.6)	11 (61.1)	0.72
Moderate HIE	5 (29.4)	7 (38.9)	0.72
Convulsion	7 (41.2)	8 (44.4)	1
IVH (II–III)	2 (11.8)	3 (16.7)	1
ICH	1 (5.9)	1 (5.6)	1
MAS	3 (17.6)	1 (5.6)	0.34
Sepsis	0	1 (5.6)	1
Inhaled NO	3 (17.6)	1 (5.6)	0.34
CHDF	2 (11.8)	2 (11.1)	1
Adrenaline	6 (35.3)	8 (44.4)	0.73
DOA	15 (88.2)	17 (94.4)	0.6
DOB	10 (58.8)	12 (66.7)	0.73
DQ ≥ 70	12 (70.6)	6 (33.3)	0.04
DQ ≥ 85	7 (41.2)	4 (22.2)	0.29
Mortality	1 (5.9)	1 (5.6)	1
During hospitalization	59 (36–179.5)	44 (28.3–312.3)	0.48

Convulsion refers to a neonatal diagnosis, not maternal history. aEEG, amplitude-integrated electroencephalography; BE, base excess; BS, burst–suppression; CAM, chorioamnionitis; CHDF, continuous hemodiafiltration; C/S, cesarean section; DOA, dopamine; DOB, dobutamine; DQ, developmental quotient; GDM, gestational diabetes mellitus; GWs, gestational weeks; HDP, hypertensive disorders of pregnancy; HELLP, hemolysis, elevated liver enzymes, low platelet count; HIE, hypoxic–ischemic encephalopathy; ICH, intracranial hemorrhage, ICH was defined as magnetic resonance imaging or head ultrasound findings of parenchymal hemorrhage, subdural/epidural hemorrhage; IVH, intraventricular hemorrhage; L/Ds, late decelerations; MAS, meconium aspiration syndrome; NO, nitric oxide; NRFS, non-reassuring fetal status; P/Ds, prolonged decelerations; PROM, premature rupture of the membranes; UA, umbilical artery; V/Ds, variable decelerations.

## Data Availability

The data presented in this study are available on request from the corresponding author. The data are not publicly available due to restrictions privacy.

## Supplementary Materials

The following supporting information can be downloaded at: https://www.mdpi.com/article/10.3390/children12050605/s1, Table S1: Institutional Protocol for ECMO Initiation during Intravascular therapeutic hypothermia.
