# Supplementary material for: Efficacy of Intravascular Therapeutic Hypothermia for Moderate to Severe Hypoxic–Ischemic Encephalopathy
Source: Children (Basel). 2025 May 6;12(5):605. doi: 10.3390/children12050605 (PMC12109600; doi:10.3390/children12050605)
Supplement: Supplementary file 1 [file children-12-00605-s001.zip › children-3570999-supplementary.pdf]

Supplementary Table S1: Institutional Protocol for ECMO Initiation during Intravascular therapeutic hypothermia

Purpose

The primary objective of intravascular therapeutic hypothermia (Intravascular cooling with ECMO) was to achieve sufficient cooling and oxygenation with minimal invasiveness in neonates presenting with moderate persistent pulmonary hypertension of the newborn.

Inclusion Criteria for ECMO Initiation

- Neonates with an oxygenation index (OI) between 25 and 40.

- Persistent hypoxemia and/or hemodynamic instability despite maximal ventilatory and pharmacological support.

- ECMO initiation was based on attending neonatologists' clinical judgment, considering the severity of hypoxemia and circulatory compromise.

Additional Clinical Parameters Considered During Patient Selection

- Cord blood pH

- Base excess

- Lactate levels

- Apgar scores at 5 and 10 minutes

Although internal cutoff values for these parameters were considered, they have not yet been validated or published. Therefore, for the purpose of this study, moderate PPHN with an OI of 25–40 was adopted as the formal inclusion criterion.

ECMO Procedure and Cooling Protocol

- ECMO was performed using a venovenous approach with low flow rates.

- Initial pump flow was set at 20 mL/kg/min and adjusted between 20–40 mL/kg/min.

- Target cooling temperature: 34.0°C, monitored at the internal jugular sinus.

- Duration of hypothermia: 72 hours.

- Rewarming protocol: gradual increase of 0.5°C per hour until reaching 37.0°C.

Contraindications for ECMO Initiation

- Presence of severe intracranial hemorrhage (e.g., Grade III–IV intraventricular hemorrhage).

- Lethal congenital anomalies or chromosomal abnormalities (e.g., trisomy 13, trisomy 18).

- Evidence of irreversible brain injury or suspected brain death.

- Severe irreversible multi-organ failure.

- Uncontrollable bleeding or severe coagulopathy.

- Determination by the attending physician that survival was not achievable even with ECMO support.

Additional Considerations

- ECMO initiation was also influenced by real-time resource availability, including ECMO machine access and availability of ECMO-trained staff.

- Institutional protocols evolved during the study period, reflecting real-world constraints in a tertiary NICU setting.

Abbreviations

ECMO: Extracorporeal Membrane Oxygenation

OI: Oxygenation Index
